# Trends in the use of non-nutritive sweeteners among female university students in the United Arab Emirates

**DOI:** 10.1016/j.pmedr.2025.103166

**Published:** 2025-07-08

**Authors:** Dalia Haroun, Aseel Ehsanallah, Wafa Mubarak

**Affiliations:** College of Natural and Health Sciences, Department of Health Sciences, Zayed University, P.O.Box 19282, Dubai, United Arab Emirates

**Keywords:** Non-nutritive sweeteners, Females, UAE, Consumption pattern

## Abstract

**Objective:**

This study aimed to explore the consumption patterns of non-nutritive sweeteners (NNS) among female university students in the United Arab Emirates (UAE), in the context of rising health awareness.

**Methods:**

A cross-sectional survey was conducted from February to April 2023 among female university students in the UAE. A total of 192 participants aged 18 and above were recruited through convenience sampling. A structured, self-administered online questionnaire was used to collect data on NNS consumption, motivations for use, physical activity, dietary habits, and self-reported height and weight. Body Mass Index (BMI) was calculated and categorized using the World Health Organization (WHO) criteria. Data were analyzed using SPSS version 22.0. Descriptive statistics and chi-squared tests were applied, with significance set at *p* < 0.05.

**Results:**

Only 24 % of participants reported current consumption of artificially sweetened foods and beverages, while 15.1 % used sweeteners in pill or powder form. The most common reasons for using NNS were weight management and adopting a healthy diet. Among former users, taste preferences (23.9 %) and perceived health risks (13 %) were primary reasons for discontinuation. Significant associations were observed between NNS use and BMI, engagement in weight loss diets, and physical activity levels.

**Conclusions:**

NNS consumption appears relatively low among female university students in the UAE, with health-driven motivations and sensory preferences shaping usage patterns. These findings highlight the need for targeted public health messaging and suggest potential shifts in dietary behavior among young women.

## Highlights

• Only 24 % used artificially sweetened foods; 15.1 % used pills or powder forms.
• Top reasons for quitting sweeteners: taste and perceived health risks.
• Health concerns were the top motivation for using non-nutritive sweeteners.
• Study offers insights into non-nutritive sweetener use among female students.

## 1 Introduction

In recent decades, rising rates of cardiovascular diseases (CVDs), type 2 diabetes, obesity, and metabolic syndrome have become major public health concerns in developed countries (Gauthier et al., 2024). In the United Arab Emirates (UAE), non-communicable diseases (NCDs) cause 55 % of deaths, with CVDs accounting for 34 % (WHO, 2021). A 2019 UAE Ministry report found acute myocardial infarction as the leading cause of CVD deaths, followed by cerebrovascular disease and hypertension (Mezhal et al., 2023). Obesity affects 27.8 % of adults (Ahmad et al., 2017-2018), and diabetes prevalence reached 12.3 % in 2021 (International Diabetes Federation, 2021). High sugar consumption is a key contributor to these trends (Andrade et al., 2021), with a 2019 study showing 56 % of UAE undergraduates are heavy added-sugar consumers (Khawaja et al., 2019). The World Health Organization (WHO) recommends reducing sugar intake to below 10 % of energy consumption (World Health Organization, 2015). In response, the food industry is incorporating non-nutritive sweeteners (NNS) to lower energy intake and glycemic index (Gauthier et al., 2024; Fowler et al., 2008).

Alternative sweeteners mimic the sweetness of sucrose and glucose-fructose syrups while providing low or zero calories (Gauthier et al., 2024; Fowler et al., 2008; Fitch and Keim, 2012). They are widely used in soft drinks, dairy products, baked goods, candies, chocolates, and as tabletop sweeteners (Gauthier et al., 2024). Their safety has been endorsed by European and international authorities, driving global consumption. The Food and Drug Administration (FDA) has approved six sweeteners: Acesulfame-K, Advantame, Aspartame, Saccharin, Sucralose, and Neotame, and recognized Stevia and monk fruit as generally safe. To ensure safe consumption, the FDA has established Acceptable Daily Intakes for NNS, such as 15 mg/kg for Acesulfame-K, 32.8 for Advantame, 50 mg/kg for Aspartame, 5 mg/kg for Saccharin, 5 mg/kg for Sucralose, and 0.3 mg/kg for Neotame (FDA, 2023).

The use of NNS has been widely promoted for its potential to address concerns related to added sugar consumption, weight loss facilitation, and blood glucose level control. Despite these perceived benefits, an array of investigations has delved into potential negative side effects associated with their usage, including concerns about insulin resistance, weight gain, cancer, and heightened appetite response (Myers et al., 2018; Sylvetsky et al., 2014). All NNS consumption has been linked to alterations in gut microbiota, contributing an additional dimension to the ongoing discussion (Gauthier et al., 2024). Although debates persist regarding the overall harmlessness of sweetener consumption on the human body, (Malek et al., 2018; Toews et al., 2019; Visek et al., 2018) there remains controversy specifically regarding NNS consumption and its impact on health, particularly in the context of weight management (Malek et al., 2018). Numerous yet-to-be-explored associations between NNS consumption and various health outcomes further contribute to the complexity of this issue (Malek et al., 2018). A general consensus on the health benefits of NNS is yet to be established and the controversy surrounding them remains evident (Mattes and Popkin, 2009; Tayel et al., 2017).

Numerous studies have examined NNS intake across populations. Data from the US National Health and Nutrition Examination Survey (2007–2012) showed 47.8 % of adults consumed more than one NNS-containing item in two days (Malek et al., 2018). In Lebanon, 94.4 % of adults had consumed artificially sweetened items in six months, mainly for weight loss or health (Daher et al., 2022). Among university students, over 80 % in Latin America consumed at least one NNS-containing food daily (Durán Agüero et al., 2017), 31 % in Egypt used NNS (Tayel et al., 2017), while 55.1 % of Caribbean students reported never using NNS (Webb et al., 2021).

However, the majority of these studies featured both male and female participants, with limited research focusing exclusively on females. Sex differences in NNS usage patterns may exist due to variations in dietary behavior, health concerns, and body image perception, which tend to differ between males and females. Studies examining women's health and weight control behaviors suggest that females are more likely than males to seek alternatives to sugar for weight management or dieting purposes, including increased use of NNS products. (Farhat et al., 2021; Drewnowski and Rehm, 2015) Although there is a growing interest in sex-specific dietary behaviors, there remains a lack of literature addressing NNS consumption specifically among females. Currently, no studies have been conducted on NNS consumption among the general population in the UAE. This study aims to investigate NNS consumption patterns among female university students.

## 2 Methods

### 2.1 Study design and sample size

This cross-sectional survey, conducted from February to April 2023, aimed to investigate the consumption of NNS among female university students in the UAE. The sample size was calculated according to the Krejcie-Morgan formula (X^2^: chi-square of a degree of freedom 1 and confidence 95 % = 3.841; N: population 4000; P: proportion of population 0.5, and e: acceptable sampling error 0.05.) (*n* = 280, *N* = 4000, e = 0.05, x2 = 3.841, *p* = 0.5). The calculated sample size was 351 females aged 18 and above, chosen through a convenient sampling setting for a quantitative cross-sectional study.

Inclusion criteria included female students enrolled in any program at the university who were 18 years or older and provided complete responses. Exclusion criteria included male students and individuals below 18 years of age. The questionnaire was self-administered and distributed online through the university's official email system and learning platforms. Participants voluntarily accessed and completed the survey, ensuring anonymity and confidentiality of responses.

### 2.2 Ethical approval

Ethical approval was authorized by the research ethics committee at Zayed University (ZU23_113_S). Prior to survey administration, all participants willingly provided informed consent. The collected data were carefully handled to ensure anonymity and confidentiality, strictly for academic purposes.

### 2.3 Measurement tools

The frequency of NNS consumption was assessed using a questionnaire adapted from Daher et al. The questionnaire comprised five sections, encompassing personal and socioeconomic status, educational level, daily habits, NNS consumption and knowledge, and anthropometric measurements (height and weight) (Daher et al., 2022). Participants were requested to answer questions regarding their experience with NNS, their usage of ingredient lists and food labels, as well as their weight loss practices. These factors were recognized as potentially confounding variables that might influence the respondents' depth of knowledge about NNS. Body Mass Index (BMI) was calculated based on self-reported data, computed by dividing weight in kilograms by the square of height in meters (kg/m^2^). Participants were then categorized into specific groups according to their BMI: underweight (<18.5 kg/m^2^), healthy weight (18.5–24.9 kg/m^2^), overweight (25–29.9 kg/m^2^), or obese (≥30 kg/m^2^), utilizing the cut-off points by WHO (World Health Organization, 2010).

Physical activity level was determined based on self-reported engagement in physical activity and categorized as follows: sedentary (no physical activity), moderately active (less than 150 min per week of moderate-intensity aerobic activity or less than 75 min per week of vigorous-intensity aerobic activity), and intensively active (more than 150 min per week). These classifications align with the physical activity guidelines provided by WHO.

The survey was piloted among five female young adults, and based on the feedback received, the questionnaire was finalized and distributed.

### 2.4 Statistical analysis

Continuous variables were reported as mean ± standard deviation, (such as age, weight, height, and BMI), while categorical variables (college, year, and knowledge) were presented as frequencies and percentages. BMI categories were grouped as underweight/normal vs. overweight/obese, merged for analysis due to small sample sizes in subgroups. NNS consumption patterns were analyzed separately for artificially sweetened foods/beverages versus pills/powders due to differing usage contexts. Chi-square (χ^2^) tests assessed associations between NNS use and: (1) BMI categories, (2) current weight-loss diet status, and (3) physical activity levels. The confidence interval (CI) was set at 95 %, and significance was considered at a *p*-value of <0.05. Data were analyzed using SPSS version 22.0.

## 3 Results

The general characteristics of the study population, consisting of 192 female university students aged 18 years and above are presented in Table 1. The majority were single (91.7 %). The participants were spread across different academic years and represented a range of colleges. Lifestyle factors, including activity levels, were assessed, whereby 46.9 % reported a moderately active lifestyle, and 29.2 % were actively engaged in weight loss diets. More than half of the participants (56.3 %) reported a history of dieting. The BMI distribution demonstrated that 58.9 % fell within the healthy weight range, and 26.6 % were overweight or obese.

**Table 1:** General characteristics of female university students in the United Arab Emirates, 2023 (*n* = 192).

	n (%)
**Living district**	136 (70.8)
Dubai	21 (10.9)
Sharjah	15 (7.8)
Umm Al Quwain	12 (6.3)
Ajman	4 (2.1)
Abu Dhabi	3 (1.6)
Ras Al-Khaimah	1 (0.5)
Marital status	
Married	16 (8.3)
Single	176 (91.7)
College	
College of Natural and Health Sciences	65 (33.9)
College of Arts and Creative Enterprises	39 (20.3)
College of Humanities and Social Sciences	28 (14.6)
College of Technological innovation	23 (12)
College of Business	18 (9.4)
College of Communication and Media Sciences	17 (8.9)
College of Interdisciplinary Studies	2 (1)
College year	
1	12 (6.3)
2	75 (39.1)
3	57 (29.7)
4	31 (16.1)
Last semester	17 (8.9)
Smoking habits	
Yes, daily	4 (2.1)
Yes, less than daily	6 (3.1)
No, not at all	182 (94.8)
Physical activity	
Sedentary	80 (41.7)
Moderately	90 (46.9)
Intensively	22 (11.5)
Currently on weight loss diet	
Yes	56 (29.2)
No	136 (70.8)
Past dieting	
Yes	108 (56.3)
No	84 (43.8)
**BMI category**	
Underweight	28 (14.6)
Normal weight	113 (58.9)
Overweight	36 (18.8)
Obese	15 (7.8)

Table 2 shows the usage patterns of NNS among participants. Among the respondents, 38.5 % have a history of using artificial sweeteners in the past. Currently, 24 % reported consuming artificial-sweetened foods and beverages, while 15.1 % used artificial sweeteners in the form of pills or powders. Additionally, 18.8 % of the participants continue to use sugar-free foods and beverages. The selection criteria for artificial sweetener-containing products varied, with 40.6 % choosing products with no specific criteria, 27.6 % refraining from their use altogether, and 15.1 % exclusively favoring one brand. In terms of perceptions, two-thirds of participants (67.7 %) expressed uncertainty about whether one type of artificial sweetener is better than another, while 17.2 % believed no type holds superiority, and 10.4 % endorsed stevia as the best among artificial sweeteners.

**Table 2:** Use of non-nutritive sweeteners among female university students in the United Arab Emirates, 2023 (n = 192).

	n (%)
*Have you used artificial sweeteners in the past?*	
No	118 (61.5)
Yes	74 (38.5)
*Do you currently consume artificially sweetened foods and beverages?*	
No	146 (76)
Yes	46 (24)
*Do you currently use artificial sweeteners in pill or powder form?*	
No	163 (84.9)
Yes	29 (15.1)
*Do you still consume sugar-free foods or beverages?*	
No	87 (45.3)
Yes	36 (18.8)
I am not using	69 (35.9)
*How do you select products containing artificial sweeteners?*	
Nothing specific	78 (40.6)
I don't use artificial sweeteners containing products	53 (27.6)
I only use one specific brand	29 (15.1)
Based on my doctor's recommendation	14 (7.3)
Based on the type of sweetener they are made of	11 (5.7)
I choose the cheapest	5 (2.6)
I choose the most expensive	2 (1.0)
*Do you believe some artificial sweeteners are better than others?*	
I don't know	130 (67.7)
No, no type is better than the other	33 (17.2)
Yes, stevia is the best	20 (10.4)
Yes, aspartame is the best	4 (2.1)
Yes, sucralose is the best	4 (2.1)
Yes, Ace-K is the best	1 (0.5)

Among current NNS users (*n* = 46 for foods and beverages; *n* = 29 for pills and powders), the motivations for usage and discontinuation are varied. As shown in Table 3, 37 % of participants reported using NNS in foods and beverages as part of a healthy diet, and 26.1 % cited weight loss as their primary motivation. In the past, 47.8 % of users consumed NNS for health-related dietary changes, while 34.8 % aimed for weight loss. For those who discontinued use, the leading reasons included disliking the taste (23.9 %) and concerns about potential harm (13 %). Family members influenced 65.2 % of current users, making them the most common source of advice, followed by dietitians (6.5 %), friends (4.3 %), and doctors (4.3 %). Among those who never used NNS, taste aversion (23.9 %), high cost (17.4 %), and perceived harm (10.9 %) were dominant reasons.

**Table 3:** Reported reasons for consuming non-nutritive sweetened foods and beverages among female university students in the United Arab Emirates, 2023 (n = 46).

	n (%)
*Why are you currently consuming foods or beverages that contain artificial sweeteners?*	
As part of a healthy diet	17 (37)
I was on a weight loss diet	12 (26.1)
I have diabetes	2 (4.3)
I have other medical condition	1 (2.2)
Other	14 (30.4)
*Why did you previously consume foods and beverages containing artificial sweeteners?*	
As part of a healthy diet	22 (47.8)
I was on a weight loss diet	16 (34.8)
I have other medical condition	2 (4.3)
Other	6 (13)
*Why did you stop consuming foods and beverages that contain artificial sweeteners?*	
I don't like their taste	11 (23.9)
I believe that they are harmful	6 (13)
I believe that there is no difference	4 (8.7)
They are expensive	4 (8.7)
Other	21 (45.7)
*Who directed you for their usage?*	
Family member	30 (65.2)
Dietitian	3 (6.5)
Friends	2 (4.3)
Doctor	2 (4.3)
Other	9 (19.6)
*Why do you choose not to consume foods and beverages that contain artificial sweeteners?*	
I don't like their taste	11 (23.9)
They are expensive	8 (17.4)
I believe that they are harmful	5 (10.9)
I believe that there is no difference	3 (6.5)
Other	19 (41.3)

In relation to pills and powders (Table 4), 41.4 % of participants currently use these as part of a healthy diet, and 27.6 % for weight loss. Former users had similar motivations, with 37.9 % and 27.6 % citing healthy diet and weight loss, respectively. Discontinuation was largely attributed to taste (17.2 %) and perceived health concerns (17.2 %). Again, family influence was dominant (55.2 %), followed by friends (20.7 %) and dietitians (10.3 %). These insights emphasize the multifaceted considerations behind NNS usage among female students and justify the inclusion of items related to past and non-use in the survey. Understanding former use and reasons for non-use provides a more comprehensive perspective on current consumption behaviors and the social and perceptual factors that may influence them.

**Table 4:** Reported reasons for using non-nutritive sweetener pills and powders among female university students in the United Arab Emirates, 2023 (n = 29).

	n (%)
*Why are you currently using pill/powder sweeteners?*	
As part of a healthy diet	12 (41.4)
I was on a weight loss diet	8 (27.6)
I have other medical condition	1 (3.4)
Other	8 (27.6)
*Why did you previously use pill/powder sweeteners in drinks or homemade desserts?*	
As part of a healthy diet	11 (37.9)
I was on a weight loss diet	8 (27.6)
I have other medical condition	2 (6.9)
Other	8 (27.6)
*Why did you stop using pill/powder sweeteners?*	
They are expensive	5 (17.2)
I believe that there is no difference	5 (17.2)
I don't like their taste	4 (13.8)
I believe that they are harmful	1 (3.4)
Other	14 (48.3)
*Who directed you for their usage?*	
Family member	16 (55.2)
Friends	6 (20.7)
Dietitian	3 (10.3)
Doctor	1 (3.4)
Other	3 (10.3)
*Why do you choose not to use pill/powder sweeteners?*	
They are expensive	7 (24.1)
I believe that there is no difference	5 (17.2)
I don't like their taste	3 (10.3)
I believe that they are harmful	2 (6.9)
Other	12 (41.4)

Table 4 shows the various factors influencing the usage of NNS in pill and powder. Currently, 41.4 % incorporate artificial sweeteners into their routine as part of a healthy diet, while 27.6 % attribute usage to weight loss. Past usage motivations include 37.9 % for a healthy diet and 27.6 % for weight loss, with 6.9 % indicating other medical conditions. Reasons for discontinuation included taste aversion (17.2 %) and a belief in potential harm (17.2 %). Family members played a significant role in influencing usage decisions, with 55.2 % seeking guidance from family, followed by friends (20.7 %) and dietitians (10.3 %). Among non-users, predominant reasons include taste aversion (23.9 %), cost considerations (17.4 %), and a dislike for taste (13.8 %).

Table 5 offers insights into the perceptions of the study population regarding NNS. Almost half of the study population (43.2 %) incorrectly believed that diet soda contained table sugar, and 28.6 % thought sugar-free products also contained table sugar. 40.6 % of participants perceived NNS as harmful, while 27.6 % mistakenly associated them with weight gain. Furthermore, 26 % mistakenly believed NNS could cause diabetes, and 22.4 % associated NNS with cancer. Perceptions about satiety and hunger varied, with 35.9 % believing that NNS-containing foods and beverages made them feel full, while 17.7 % thought they induced hunger.

**Table 5:** Perceptions on non-nutritive sweeteners among female university students in the United Arab Emirates, 2023 (n = 192).

	n (%)
*Do you believe diet soda contain table sugar?*	
Yes	83 (43.2)
No	51 (26.6)
I don't know	58 (30.2)
*Do you believe sugar-free products contain table sugar?*	
Yes	55 (28.6)
No	75 (39.1)
I don't know	62 (32.3)
*Do you consider artificial sweeteners harmful?*	
Yes	78 (40.6)
No	57 (29.7)
I don't know	57 (29.7)
*Do you believe artificial sweeteners cause weight gain?*	
Yes	53 (27.6)
No	63 (32.8)
I don't know	76 (39.6)
*Do you believe artificial sweeteners cause diabetes?*	
Yes	50 (26)
No	71 (37)
I don't know	71 (37)
*Do you believe artificial sweeteners contain calories?*	
Yes	86 (44.8)
No	53 (27.6)
I don't know	53 (27.6)
*Do you believe artificial sweeteners cause cancer?*	
Yes	43 (22.4)
No	61 (31.8)
I don't know	88 (45.8)
*Do you believe foods and beverages containing artificial sweeteners make you feel full?*	
Yes	69 (35.9)
No	45 (23.4)
I don't know	78 (40.6)
*Do you believe foods and beverages containing artificial sweeteners make you feel hungry?*	
Yes	34 (17.7)
No	65 (33.9)
I don't know	93 (48.4)

Table 6 presents the relationship between the usage of artificially sweetened food and beverages and pills and powders with BMI categories, being on a weight loss diet, and physical activity levels. BMI categories were merged underweight/normal and overweight/obese due to the small sample in the different categories. The rate of using artificially sweetened food and beverages was significantly higher among individuals categorized as overweight/obese (35.3 %) compared to those classified as under/normal weight (19.9 %); *p* = 0.03. However, no significant difference was found between the usage of artificial sweetener pills and powders among the different BMI categories (*p* = 0.55). Among users of artificially sweetened food and beverages, 37.5 % are on a weight loss diet, significantly higher than the 18.4 % who are not (*p* = 0.01). No significant difference was found among users of artificial sweetener pills and powders (*p* = 0.26) and being on a weight loss diet. The results also revealed that among users of artificially sweetened food and beverages, 40.9 % were physically active. Similarly, among users of artificial sweetener pills and powders, 31.8 % live an active lifestyle.

**Table 6:** Association between non-nutritive sweetener use and body mass index, engagement in weight loss diets, and physical activity levels among female university students in the United Arab Emirates, 2023 (n = 192).

Foods and Beverages				
	Users (n = 46) n (%)	Non-users (*n* = 146) n (%)	Total (*n* = 192)	P-value
*BMI category*				
Under/normal weight	28 (19.9)	113 (80.1)	141	0.03
Overweight/obese	18 (35.3)	33 (64.7)	51	
*Currently on weight loss diet*				
Yes	21 (37.5)	35 (62.5)	56	0.01
No	25 (18.4)	111 (81.6)	136	
*Physical activity level*				
Sedentary	9 (11.3)	71 (88.8)	80	0.00
Moderate	28 (31.1)	62 (68.9)	90	
Active	9 (40.9)	13 (59.1)	22	

*Pills and Powders*				
	Users (n = 46) n (%)	Non-users (*n* = 163) n (%)	Total (n = 192)	P-value
*BMI category*				
Under/normal weight	20 (14.2)	121 (85.8)	141	0.55
Overweight/obese	9 (17.6)	42 (82.3)	51	
*Currently on weight loss diet*				
Yes	18 (13.2)	118 (86.8)	136	0.26
No	11 (19.6)	45 (80.4)	56	
*Physical activity level*				
Sedentary	8 (10)	72 (90)	80	0.04
Moderate	14 (15.5)	76 (84.4)	90	
Active	7 (31.8)	15 (68.2)	22	

Associations between non-nutritive sweetener (NNS) usage and BMI category, weight loss diet, and physical activity levels were assessed using the Chi-square test. A p-value <0.05 was considered statistically significant.

## 4 Discussion

The present study aimed to investigate NNS consumption patterns among female university students in the UAE. In terms of artificial sweetener usage patterns, this study found that only 24 % reported consuming artificial-sweetened foods and beverages, while 15.1 % used artificial sweeteners in the form of pills or powders. Similarly, a 2022 Food and Health Survey in the US showed a preference for sugar over NNS, with 31 % favoring sugar compared to 24 % for sweeteners. Interestingly, younger generations are more inclined to use NNS in the US (Food and Health Survey, 2022). In contrast, a study conducted in Lebanon by Daher et al. found a much higher usage rate, with 94.1 % reporting consumption of food and beverages containing NNS (Daher et al., 2022). The fact that 45.3 % of participants in this study continue not to use sugar-free foods and beverages suggests a potential shift in consumption habits or increased awareness of artificial sweetener-containing products in recent times. Likewise, it was reported that nearly 73 % of Americans are actively attempting to limit or avoid sugars altogether, indicating a widespread trend toward reducing sugar intake (Food and Health Survey, 2022).

Among those who stopped using artificial sweeteners, common reasons included taste preferences and perceived harm, consistent with findings from Lebanon and the US where safety concerns and unpleasant taste led to discontinuation (Food and Health Survey, 2022). In Egypt, university students also cited undesirable taste as the main side effect of NNS use (Tayel et al., 2017). A study by Tan et al. found Acesulfame-K, stevia, and monk fruit had strong, lingering off-tastes with less sweetness, while aspartame and sucralose had bitter, metallic flavors despite maintaining sweetness (Tan et al., 2019). Regarding perceived harm, a systematic review and meta-analyses found that across a broad range of benefits and harms of NNS in a generally healthy population of adults and children, there were no statistically or clinically relevant differences observed between NNS intake versus no intake, or between different doses of NNS for most outcomes. Therefore, the health effects of NNS consumption are still inconclusive (Andrade et al., 2021; Toews et al., 2019).

Stevia was the most recognized sweetener among participants (53.6 %), suggesting greater exposure compared to others. Higher awareness of sweeteners like Stevia may encourage usage, while lower awareness of Acesulfame-K may limit it. In the US, sugar, honey, and maple syrup are preferred, followed by Aspartame, Sucralose, and Stevia (Food and Health Survey, 2022). In contrast, Tayel et al. found Sucralose and Aspartame were most used by Egyptian university students (Tayel et al., 2017). Webb et al. reported many Caribbean students were unaware of NNS-related health risks, believing NNS aid weight loss (Webb et al., 2021). In South Africa, adults expressed safety concerns, particularly cancer risks, and showed interest in learning more about NNS to make informed health decisions (World Health Organization, 2022; Naicker et al., 2024).

The results of this study revealed that the primary reason behind the usage of NNS is health-related considerations. Participants who incorporate NNS into their diet do so as part of a strategy for maintaining a healthy diet and managing weight loss. This aligns with findings from the US, where the primary reason for NNS usage was to manage diabetes or regulate blood sugar levels. Other motivations included reducing carbohydrate intake for weight maintenance or weight loss and conserving calories to indulge in other food or beverage options (Food and Health Survey, 2022). Similarly, in Lebanon, individuals reported using NNS primarily for weight loss purposes, followed by a combination of reasons such as adopting a healthy lifestyle and enjoying the favorable taste (Daher et al., 2022). In Egypt as well, artificial sweeteners were used by 31 % of Alexandria University students and were most common among females who were mostly following a diet regimen that necessitated the restriction of sugar intake (Tayel et al., 2017). Other studies have also reported that females tend to be more concerned about their weight and caloric intake than males, opting to use NNS instead of sugar for caloric control (Malek et al., 2018; Daher et al., 2022; Drewnowski and Rehm, 2015; González-Rodríguez et al., 2021).

The study found a significant association between being categorized as overweight/obese and the usage of artificially sweetened food and beverages, with a higher rate of usage among individuals in this BMI category compared to those classified as under/normal weight. This suggests that individuals with higher BMI may be more likely to consume NNS as part of their dietary habits. Similar results were seen in the UK, it was noted that individuals classified as overweight or obese tend to consume NNS more commonly. This usage pattern suggests that NNS might be utilized for weight management purposes, given their low-calorie content, availability across various food and beverage choices, and ability to satisfy food cravings (Farhat et al., 2021). This study also found a significant association between being on a weight loss diet and the usage of artificially sweetened food and beverages, with a higher rate of usage among individuals on weight loss diets compared to those not on such diets. This corresponds with the results of Tayel et al., indicating that individuals who consume artificial sweeteners are significantly more likely to follow a dietary regimen compared to those who do not use them (Tayel et al., 2017).

This study has several limitations, including the use of convenience sampling and a small sample size, which may introduce selection bias and limit the generalizability of the findings. While the sample size was calculated according to the Krejcie-Morgan formula, the actual sample size achieved was 192, failing to meet the calculated sample size of 351, resulting in a percent error of 82.81 % and potentially impacting the precision of the study's findings. Additionally, the study focused exclusively on female university students, thus not providing insights into NNS consumption patterns among males in the UAE. Due to the cross-sectional design, the study could not establish a causal relationship between variables. Future research could explore the main sources of NNS, and the quantity consumed to provide a more comprehensive understanding of NNS intake patterns.

## 5 Conclusion

This study reveals relatively low NNS usage among female university students in the UAE, primarily motivated by weight management and health considerations, while taste preferences and safety concerns contribute to discontinuation. Persistent knowledge gaps about NNS health effects highlight the need for targeted, evidence-based nutrition guidance for young adults in the UAE.

## Research data

Due to the sensitive nature of the questions asked in this study, survey respondents were assured raw data would remain confidential and would not be shared.

## CRediT authorship contribution statement

**Dalia Haroun:** Supervision, Formal analysis, Conceptualization. **Aseel Ehsanallah:** Writing – original draft, Formal analysis. **Wafa Mubarak:** Methodology, Investigation, Formal analysis.

## Ethical approval

Ethical approval was obtained from Zayed University Research Ethics Committee (ZU23_113_S).

## Funding

This research did not receive any specific grant from funding agencies in the public, commercial, or not-for-profit sectors.

## Declaration of competing interest

The authors declare that they have no known competing financial interests or personal relationships that could have appeared to influence the work reported in this paper.

## Data Availability

Data will be made available on request.
